# *OTOF* mutation screening in Japanese severe to profound recessive hearing loss patients

**DOI:** 10.1186/1471-2350-14-95

**Published:** 2013-09-22

**Authors:** Yoh-ichiro Iwasa, Shin-ya Nishio, Hidekane Yoshimura, Yukihiko Kanda, Kozo Kumakawa, Satoko Abe, Yasushi Naito, Kyoko Nagai, Shin-ichi Usami

**Affiliations:** 1 Department of Otorhinolaryngology, Shinshu University School of Medicine, 3-1-1 Asahi, Matsumoto, Nagano 390-8621, Japan; 2 Kanda ENT Clinic, 4-25 Wakakusa-cho, Nagasaki 852-8023, Japan; 3 Department of Otorhinolaryngology, Toranomon Hospital, 2-2-2 Toranomon, Minato-ku, Tokyo 105-8470, Japan; 4 Department of Otolaryngology, Kobe City Medical Center General Hospital, 2-1-1 Minatojima Minamimachi, Chuou-ku, Kobe City 650-0047, Japan; 5 Department of Otorhinolaryngology, Gunma University School of Medicine, 3-39-15 Shouwa-machi, Maebashi, Gunma 371-8511, Japan

**Keywords:** Auditory neuropathy spectrum disorder, DFNB9, Nonsyndromic hearing loss

## Abstract

**Background:**

Auditory neuropathy spectrum disorder (ANSD) is a unique form of hearing loss that involves absence or severe abnormality of auditory brainstem response (ABR), but also the presence of otoacoustic emissions (OAEs). However, with age, the OAEs disappear, making it difficult to distinguish this condition from other nonsyndromic hearing loss. Therefore, the frequency of ANSD may be underestimated. The aim of this study was to determine what portion of nonsyndromic hearing loss is caused by mutations of *OTOF*, the major responsible gene for nonsyndromic ANSD.

**Methods:**

We screened 160 unrelated Japanese with severe to profound recessive nonsyndromic hearing loss (ARNSHL) without *GJB2* or *SLC26A4* mutations, and 192 controls with normal hearing.

**Results:**

We identified five pathogenic *OTOF* mutations (p.D398E, p.Y474X, p.N727S, p.R1856Q and p.R1939Q) and six novel, possibly pathogenic variants (p.D450E, p.W717X, p.S1368X, p.R1583H, p.V1778I, and p.E1803A).

**Conclusions:**

The present study showed that *OTOF* mutations accounted for 3.2–7.3% of severe to profound ARNSHL patients in Japan. *OTOF* mutations are thus a frequent cause in the Japanese deafness population and mutation screening should be considered regardless of the presence/absence of OAEs.

## Background

Auditory neuropathy (AN), a unique form of hearing loss, involves absence or severe abnormality of auditory brainstem response (ABR), but presence of otoacoustic emissions (OAE) and/or cochlear microphonic (CM). This disorder was defined by Starr [1], and also reported as “Auditory nerve disease” [2] and “Auditory dys-synchrony” [3]. AN was renamed “auditory neuropathy spectrum disorder (ANSD)” in 2008, due to the heterogeneous and multifaceted nature [4].

The prevalence of ANSD in sensorineural hearing loss is reported to be 0.5-15% [5]. The etiologies of ANSD are various; patients range from infants to adults, 42% of which are associated with hereditary neurological disorders, 10% with toxic, metabolic, immunological and infectious causes, and 48% with unknown causes [6]. Although the exact percentage of nonsyndromic ANSD is unclear, responsible genes have been gradually revealed. To date, mutations of *AUNA1, OTOF, PJVK, GJB2* and mitochondrial 12S rRNA are reported to be causal for nonsyndromic ANSD [7].

The *OTOF* gene (DFNB9) is mainly expressed in cochlear inner hair cells, and is necessary for synaptic exocytosis at the auditory ribbon synapse [8]. It encodes both long and short isoforms with the long isoform containing six C2 domains and the C-terminal transmembrane domain, and the short isoform containing only the last three C2 domains [9]. Mutations in the *OTOF* gene, encoding otoferlin, are reported to be the major causes of nonsyndromic recessive ANSD [10-12]. In Japanese, mutations in *OTOF* account for 56. 5% (13/23) of ANSD [13]. Although ANSD can be characterized by the presence of OAEs in the first two years of life, OAEs later disappear and the hearing loss then resembles other types of nonsyndromic hearing loss [14]. Because of expected good outcomes of cochlear implantation for patients with *OTOF* mutations [15,16], it is important to perform mutation screening for *OTOF* to select the appropriate intervention. Although some reports have described *OTOF* mutations in severe to profound autosomal recessive hearing loss patients in other populations [11,12], there has been no literature available regarding the screening of *OTOF* mutations using a large cohort in a comprehensive manner. The goal of this study was therefore to reveal the frequency of ANSD and to identify *OTOF* mutations in Japanese ARNSHL patients.

## Methods

### Subjects

Among the 1511 Japanese independent hearing loss patients registered in our DNA sample bank, 469 were congenital severe to profound sensorineural hearing loss (above 71 dB average over 500, 1000, 2000 and 4000 Hz in the better hearing ear) patients compatible with autosomal recessive inheritance (including sporadic cases). From those, we randomly selected 160 patients. All ANSD cases were sporadic (compatible with autosomal recessive inheritance). They were diagnosed as ANSD by evaluation of OAE response. We excluded autosomal dominant families because in previous studies *OTOF* mutations were not found in such groups [17]. Pure tone audiometry was used for adults (N= 32) and ABR, auditory steady-state responses (ASSR), and conditioned orientation response audiometry (COR) were used for pediatric patients (n=128). The control group was composed of 192 unrelated Japanese individuals who had normal hearing shown by auditory testing. All subjects gave prior informed written consent for participation in the project and the Ethical Committee of Shinshu University approved the study.

### Mutation analysis

We designed 43 pairs of primers to amplify DNA fragments containing all exons in the coding regions of the *OTOF* gene (ENST00000403946). Primer3Plus (http://www.bioinformatic.nl/cgi-bin/primer3plus/primer3plus.cgi) was used to design primers to flank all the exon-intron boundaries. Each genomic DNA sample (40 ng) was amplified, using Ampli Taq Gold (Applied Biosystems, Foster City, CA), for 5 min at 95°C, followed by 30 three-step cycles of 95°C for 30s, 60°C for 30s, and 72°C for 60s, with a final extension at 72°C for 7 min, ending with a holding period at 4°C in a PCR thermal cycler (Takara, Shiga, Japan). PCR products were treated with ExoSAP-IT® (GE Healthcare Bio, Santa Clara, CA) by incubation at 37°C for 60 min, and inactivation at 80°C for 15 min. After the products were purified, we performed standard cycle-sequencing reactions with ABI Big Dye® terminators in an ABI PRISM 3100 Genetic Analyzer autosequencer (Applied Biosystems, Foster City, CA).

Computer analysis to predict the effect of missense variants on the protein function was performed with wANNOVAR [18-20] (http://wannovar.usc.edu) including functional prediction software listed below. PhyloP (http://hgdownload. cse.ucsc.edu/goldenPath/hg18/phyloP44way/), Sorting Intolerant from Tolerant (SIFT; http://sift.jcvi.org/), Polymorphism Phenotyping (PolyPhen2; http://genetics.bwh.harvard.edu/pph2/), LRT (http://www.genetics.wustl.edu/jflab/lrt_query.html), and MutationTaster (http:// www.mutationtaster.org/).

## Results

**Figure 1:**
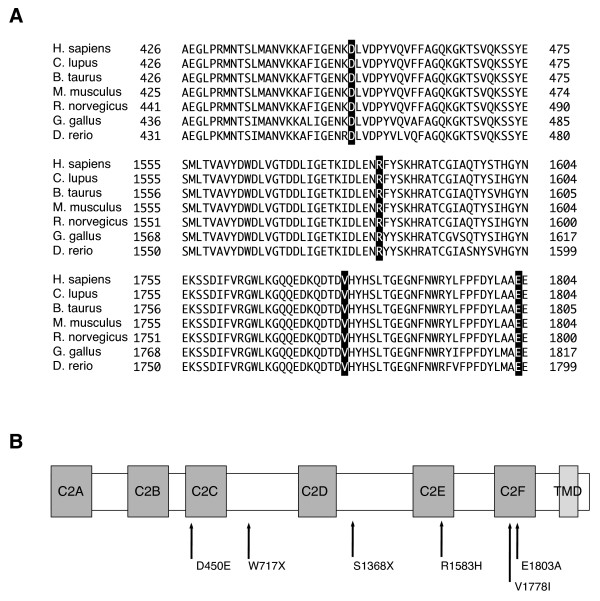
**The location of mutations in otoferlin protein and the evolutionary conservation of the amino acids. (A)** Evolutionary conservation. The locations of mutations are boxed. **(B)** Novel pathogenic *OTOF* mutations found in this work and relation to the functional domains of otoferlin. C2A-F: C2 domains. TMD: transmembrane domain.

We found a total of 11 probable pathogenic variants in the patients (Table 1). Among them, five mutations were previously reported: p.D398E, p.Y474X, p.N727S, p.R1856Q and p.R1939Q. The other six probable pathogenic variants were novel: 2 nonsense mutations (p.W717X, p.S1368X) and 4 missense mutations (p.D450E, p.R1583H, p.V1778I, p. E1803A). Based on the prediction programs, it is most likely that p.D450E (c.1350C>G), p.R1583H (c.4748G>A), p.V1778I (c.5332G>A), and p.E1803A (c.5408A>C) were pathogenic. In addition, they were absent (or in very few numbers) in the controls, and located in C2 domains, which are highly conserved among species (Figure 1). In addition, polymorphic changes were also identified (Table 2). p.R1676C (c.5026C>T) was previously reported to be pathogenic [21], but we excluded p.R1676C as it is unlikely to be pathologic because of high frequencies in the control population (Table 2). Among the 16 patients with *OTOF* mutations, 4 were homozygous, 3 were compound heterozygotes, and 9 were heterozygous without second mutation (Table 3). After clinical re-evaluation, we recategorized cases with OAE as ANSD.

**Table 1:** Probable pathogenic and uncertain pathogenic variants of OTOF identified in this study

**Exon**	**DNA level**	**Protein level**	**Occurrence in this work (chromosome)**	**Control (chromosome)**	**Functional prediction**						**References**
					**PhyloP**	**SIFT (p-value)**	**P2 D.S.**	**LRT**	**Mutation taster**	**GERP ++**	
Probable pathogenic variants											
Exon 14	c.1422T>A	p.Y474X	2/320	0/374	N (0.072941)	NA (0.829813)	NA (0.58309)	D (1)	A (1)	−3.78	[13]
Exon 18	c.2151G>A	p.W717X	1/320	0/344	C (0.994764)	NA (0.90345)	NA (0.734698)	D (0.999998)	A (1)	3.83	This study
Exon 34	c.4103C>G	p.S1368X	1/320	0/364	N (0.944413)	NA (0.915)	NA (0.554899)	NA (0.026679)	A (1)	0.571	This study
Exon 38	c.4748G>A	p.R1583H	1/320	0/366	C (0.997935)	D (1)	D (0.999)	D (1)	D (0.999661)	4.69	This study
Exon 44	c.5567G>A	p.R1856Q	1/320	0/380	C (0.99611)	T (0.91)	P (0.813)	D (1)	D (0.999517)	4.1	[11]
Exon 46	c.5816G>A	p.R1939Q	11/320	0/382	N (0.996658)	T (0.92)	NA (0.746672)	NA (1)	D (0.999886)	1.38	[22]
Uncertain pathogenic variants											
Exon 12	c.1194T>A	p.D398E*	1/320	1/380	N (0.232793)	T (0.77)	D (0.853)	D (1)	D (0.995165)	0.981	[23]
Exon 13	c.1350C>G	p.D450E*	1/320	1/380	C (0.986229)	T (0.74)	D (0.853)	D (1)	D (0.991594)	3.54	This study
Exon 18	c.2180A>G	p.N727S*	2/320	1/344	C (0.992986)	T (0.27)	P (0.386)	D (1)	D (0.95528)	3.98	[21]
Exon 43	c.5332G>A	p.V1778I	1/320	0/378	C (0.997116)	T (0.54)	P (0.289)	D (1)	D (0.994783)	4.38	This study
Exon 43	c.5408A>C	p.E1803A	1/320	0/378	C (0.994555)	D (1)	D (0.995)	D (1)	D (0.999914)	4.26	This study

*the variants found in controls.

Exon number was named based on ENST00000403946.

A, disease causing automatic; C, conserved; D, damaging or disease causing; N, not conserved; NA, not applicable; P, possibly damaging; T, tolerated; P2 D.S., Polyphen-2 damaging score. Polyphen-2, PhyloP, LRT, Mutation Taster, and GERP++ are functional prediction scores that indicate a probable mutation with increasing value.

**Table 2:** Non-pathogenic variants of OTOF identified in this study

**Exon**	**DNA level**	**Protein level**	**Occurrence in this work (chromosome)**	**Control (chromosome)**	**References**
Exon 3	c.145C>T	p.R49W	5/320	10/238	[13]
Exon 3	c.157G>A	p.A53T	2/320	3/238	[23,24]
Exon 3	c.158C>T	p.A53V	42/320	110/238	[23]
Exon 4	c.244C>T	p.R82C	14/320	27/376	[23]
Exon 21	c.2452C>T	p.R818W	1/320	3/356	[12]
Exon 40	c.5026C>T	p.R1676C	1/320	3/356	[21]

**Table 3:** Patients who have at least one pathogenic mutation identified in this study

**Patient**	**DNA level**	**Protein level**	**Clinical diagnosis**	**OAE**	**Age at diagnosis**	**Hearing loss level**
1	c.1422T>A / c.5567G>A	p.Y474X / p.R1856Q	ANSD	+	1y6m	Profound
2	c.1422T>A / c.5816G>A	p.Y474X / p.R1939Q	ANSD	+	NA	Profound
3	c.5816G>A / c.5816G>A	p.R1939Q / p.R1939Q	ANSD	+	4m	Profound
4	c.5816G>A / c.5816G>A	p.R1939Q / p.R1939Q	ANSD	+	10m	Profound
5	c.5816G>A / c.5816G>A	p.R1939Q / p.R1939Q	ANSD	+	NA	Profound
6	c.4748G>A / c.5816G>A	p.R1583H / p.R1939Q	NSHL	NA	6m	Profound
7	c.2151G>A / c.5816G>A	p.W717X / p.R1939Q	NSHL	-	1y4m	Profound
8	c.5816G>A / -	p.R1939Q /-	ANSD	+	1y5m	Profound
9	c.5816G>A / -	p.R1939Q /-	ANSD	+	7m	Profound
10	c.1194T>A / -	p.D398E / -	NSHL	NA	NA	Profound
11	c.1350C>G / -	p.D450E / -	NSHL	NA	2y	Severe
12	c.2180A>G / -	p.N727S / -	NSHL	NA	6m	Profound
13	c.2180A>G / -	p.N727S / -	NSHL	NA	1y	Severe
14	c.4103C>G / -	p.S1368X / -	NSHL	NA	7m	Profound
15	c.5332G>A / -	p.V1778I / -	NSHL	NA	NA	Profound
16	c.5408A>C / -	p.E1803A / -	NSHL	NA	4m	Profound

*ANSD* Auditory neuropathy spectrum disorder, *NSHL* Nonsyndromic sensorineural hearing loss.

## Discussion

So far, more than 90 pathologic mutations have been reported in *OTOF*[25]. The present study identified 11 possibly pathogenic *OTOF* variants in Japanese patients with nonsyndromic hearing loss, and 6 of them were novel mutations (p.D450E, p.W717X, p.S1368X, p.R1583H, p.V1778I, and p.E1803A). Concerning pathogenicity of the four novel missense mutations, p.R1583H is more likely to be a disease causing mutation, because 1) it was found in compound heterozygosity with p.R1939Q, 2) it was absent in controls, 3) it affects a C2 domain, and 4) the scores provided by prediction programs also agree with the pathogenicity. The pathogenic potential of the three other variants (p.D450E, p.V1778I, and p.E1803A) is less clear, because 1) all of them have been found in the heterozygous state without accompanying mutation in the other allele, and 2) p.D450E was found in controls. But it is also true that 1) they affect C2 domains, and 2) the scores of the prediction programs would support their classification as pathogenic variants.

As with other genes, the spectrum of *OTOF* mutations found in the Japanese population was quite different from those reported in Caucasians [13,26-28].

With regard to recurrent mutations, p.Q829X especially has a high frequency in Spanish people, being present in about 3% of all cases of recessive prelingual deafness [24]. C.2905-2923delinsCTCCGAGCGGCA is also common in Argentineans [12] and p.E1700Q is reported to be frequent in Taiwanese [29]. p.R1939Q, previously identified in the United States [22] and most recently reported as a frequent mutation in Japanese [13], was also frequently found in this study. Among 160 patients, 8 (5.0%) had this mutation, confirming it is indeed a recurrent mutation in Japanese.

Those recurrent mutations have been proved to be due to founder effects [13,24,29].

Out of 16 patients with *OTOF* mutations, 7 showed ANSD phenotype, confirming that *OTOF* mutations are major causes of ANSD. In this study, 9 were heterozygous without second mutation. A hallmark of recessive mutations is the detection of two mutations in the paternal and maternal alleles and the parents having normal hearing. As seen in previous mutation screening reports, including those for *OTOF*[12,23,30], there were a significant number of heterozygous cases without a second mutation even after direct sequencing of the coding region of the gene. Possible explanations are: 1) the existence of a second mutation in the intron or regulatory region of *OTOF*, which has not been explored, 2) the existence of a large deletion [31], 3) contribution to hearing loss by an additional modulatory gene, and 4) the existence of a mutation in another gene and just coincidental carrying of the *OTOF* mutation.

As seen in Table 3, two heterozygous patients (#8, 9) having the ANSD phenotype, are most likely to have *OTOF* related deafness.

It is assumed that *OTOF* mutations accounted for deafness in at least 7, and possibly 16, of the 160 patients (4.4-10.0%). As described in the subject section, we excluded the subjects carrying *GJB2* and *SLC26A4* mutations*.* We also excluded another responsible gene (*PJVK*), because no mutations in this gene were found. Since the frequencies of *GJB2* and *SLC26A4* gene mutations among the patients with nonsyndromic severe to profound congenital SNHL are 27.0% based on our database, mutation frequency of *OTOF* among the total of severe to profound recessive nonsyndromic SNHL is considered to be about 3.2-7.3% (which is calculated by ((7-16)/160×(100/73))×100%). Although simple comparison regarding frequency is difficult because of sampling bias, it is estimated that the frequency of OTOF mutations in Japanese may be almost equal to other populations, as mutation frequency of OTOF was reported at 2.3% (13/557) in Pakistanis [11], 5.0% in Turkish [32], 1.4% (1/73) in Chinese [23], and 18.2% (4/22) in Taiwanese [29], and 3.2% (23/708) in Spanish [12]. Although simple comparison regarding frequency is difficult because of sampling bias, it is estimated that the frequency of *OTOF* mutations in Japanese may be almost equal to other populations. In Japanese, *GJB2, SLC26A4*, *CDH23* and the 1555A>G mutation in the mitochondrial 12S rRNA are the major causes of hearing loss [33]. Considering the frequency, the *OTOF* gene may be one of the candidate genes to be screened for recessive severe to profound recessive SNHL.

The benefits of cochlear implantation for patients with ANSD has varied [34,35], but implantation has been shown to be effective for the patients with *OTOF* mutations [15,16,36], because their auditory nerves and spiral ganglions are preserved. Consequently, if an *OTOF* mutation is identified in a deaf patient, we can anticipate a good outcome of cochlear implantation, therefore, it is important and meaningful to identify genetic mutations in patients.

Most patients with *OTOF* mutations have a phenotype of stable prelingual and severe to profound nonsyndromic hearing loss. On the other hand, other phenotypes have also been reported. For example, a Taiwanese patient with an p.E1700Q mutation displayed moderate to profound progressive hearing loss [29]. Temperature sensitive ANSD, a particular form of ANSD, has also been reported in some populations [10,23,37].

In the very young child, electrophysiological testing may indicate that *OTOF*-related deafness is ANSD, but by age two OAEs have generally disappeared and the test results are more in accord with the findings of cochlear lesions [14]. Therefore, if OAE is not tested at a very early age, patients with *OTOF* mutations are not deemed to have ANSD (i.e., hidden ANSD). In fact, 9 out of our 16 patients were diagnosed genetically as nonsyndromic sensorineural hearing loss (NSHL). According to the present data, screening for *OTOF* is necessary not only for the patients diagnosed with ANSD, but also should be extended to ARNSHL cases. The current data indicated that OAE testing must always be conducted in addition to ABR in infants. And we should bear in mind that there may be patients with *OTOF* mutations among the patients diagnosed as having ARNSHL.

## Conclusions

The present study showed that *OTOF* mutations accounted for 3.2-7.3% of recessive severe to profound SNHL patients in Japan. *OTOF* mutations are a frequent cause in the Japanese deafness population and mutation screening should be considered regardless of the presence/absence of OAEs.

## Competing interests

The authors declare that they have no competing interests.

## Authors' contributions

YI and SN carried out the molecular genetic studies and the sequence alignment, and participated in drafting the manuscript. SU conceived of the study, and participated in its design and coordination and helped to draft the manuscript. All authors read and approved the final manuscript.

## Pre-publication history

The pre-publication history for this paper can be accessed here:

http://www.biomedcentral.com/1471-2350/14/95/prepub
